# Supplementary figures and images for: A pair of ascending neurons in the subesophageal zone mediates aversive sensory inputs-evoked backward locomotion in Drosophila larvae
Source: PLoS Genet. 2020 Nov 2;16(11):e1009120. doi: 10.1371/journal.pgen.1009120 (PMC7605633; doi:10.1371/journal.pgen.1009120)

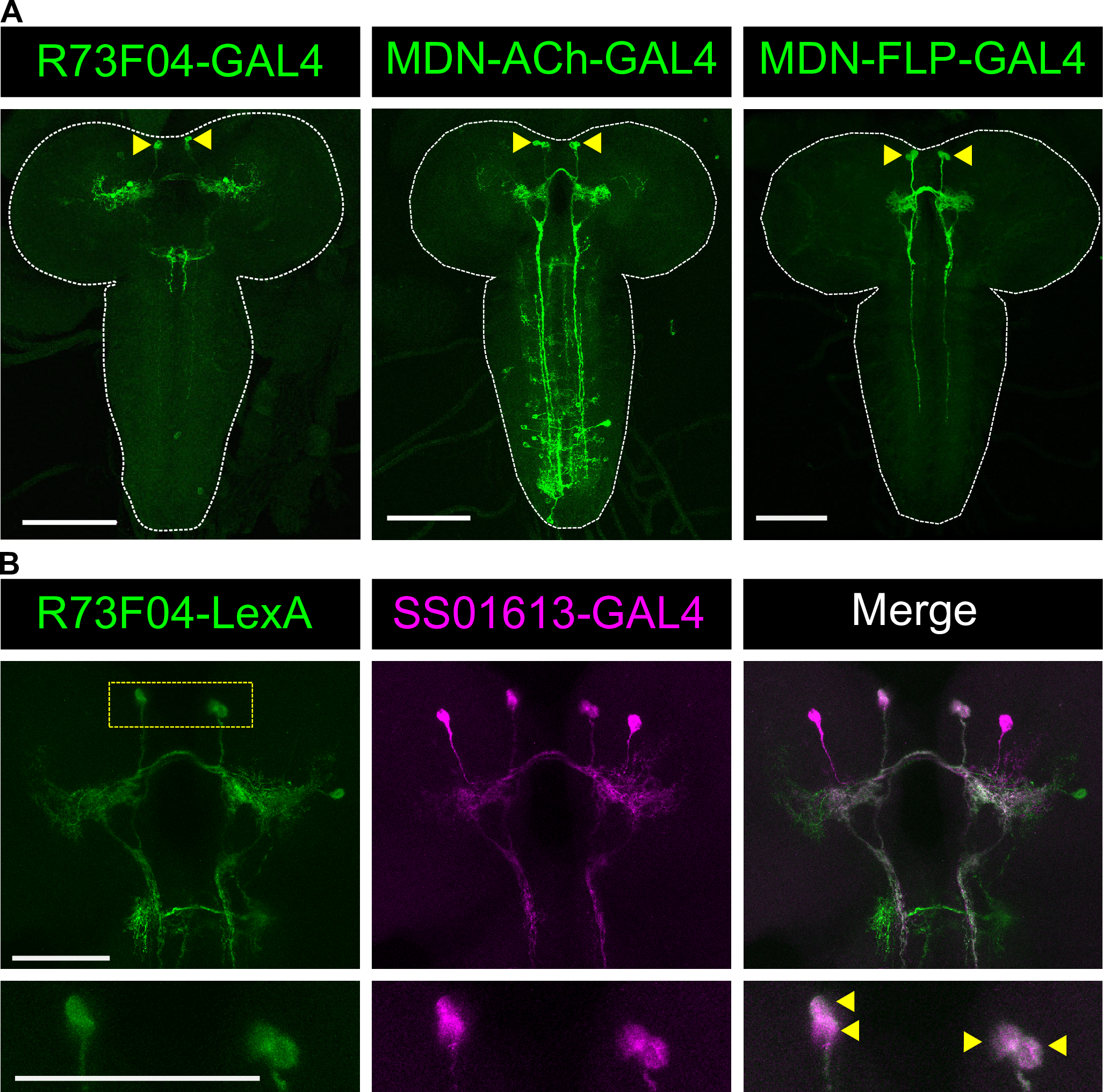

Supplement: S1 Fig — (A) Expression pattern of MDN-labeling GAL4 used in this study. The yellow arrowheads indicate the soma of MDNs. Genotypes: w; UAS-mCD8GFP, tsh-GAL80/+; R73F04-GAL4/+(R73F04-GAL4); w; UAS-mCD8GFP, tsh-GAL80/+; R73F04-GAL4, Gad1-2A-GAL80/+ (MDN-ACh); w; UAS-mCD8GFP/Otd-FLP, tub-FRT-GAL80-FRT; R73F04-GAL4, Gad1-2A-GAL80/+ (MDN-FLP). Scale bar, 100 μm. (B) Dual-labeling with MDN-labeling GAL4 used in the previous study [7] and R73F04-LexA. The yellow dot square in the upper row indicates the area shown in the lower row. The yellow arrowheads indicate both R73F04-LexA and SS01613-GAL4 label the soma of MDNs. Scale bar, 50 μm. (TIF) [file pgen.1009120.s001.tif]

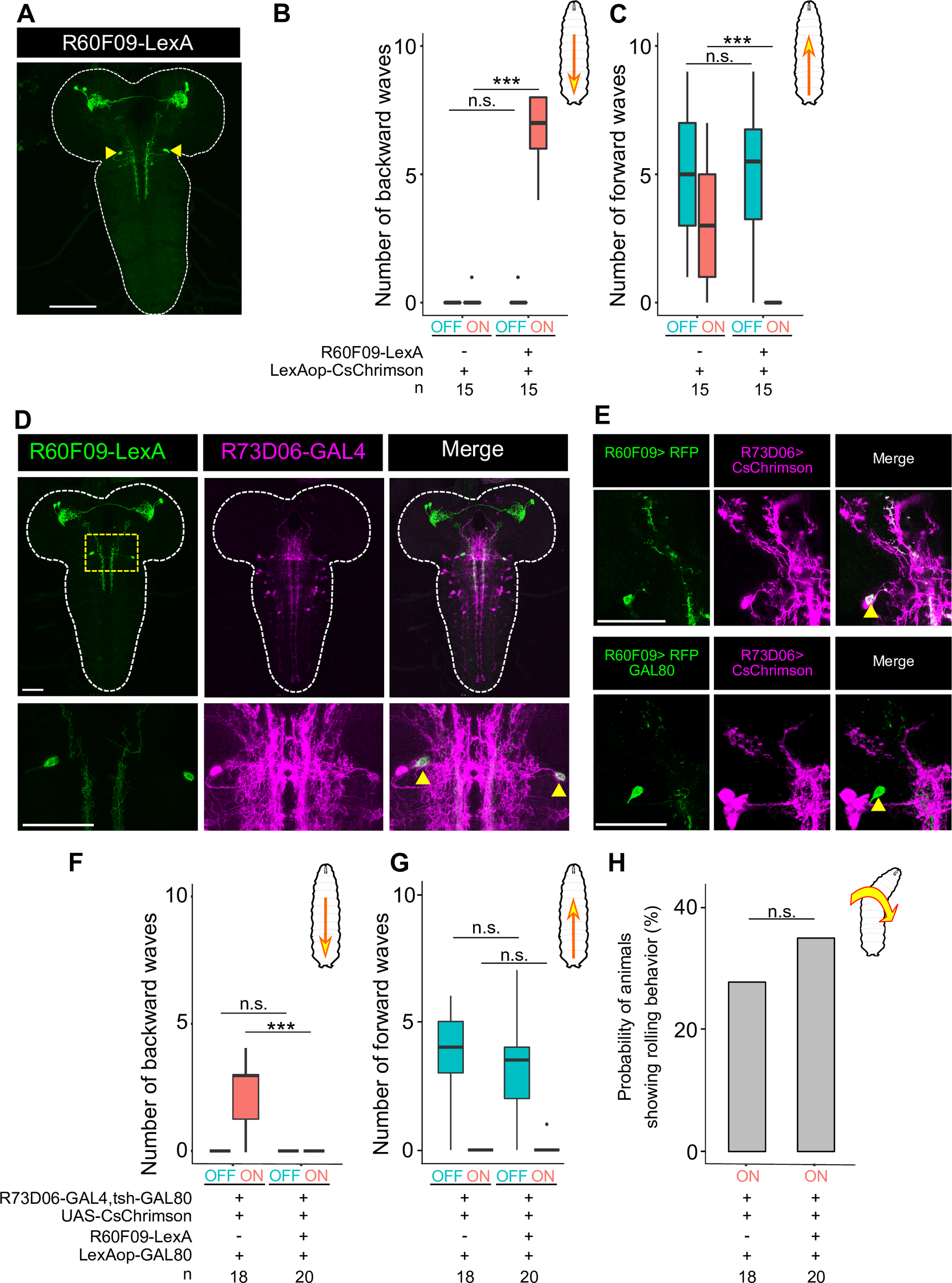

Supplement: S2 Fig — Two distinct GAL4 lines label AMBs (A) Expression patterns of R60F09-LexA in the third instar larval CNS. The yellow arrowheads indicate the soma of AMBs. Maximum intensity projection of the entire CNS shown. Genotypes: LexAop-mCD8GFP, UAS-mCD8RFP/+; R60F09-LexA/+; +/+. Scale bar, 100 μm. (B, C) The number of backward/forward waves in 10 seconds before (OFF) or during (ON) optogenetic activation with CsChrimson. n = 15 for each genotype. We assessed statistical significance by the Wilcoxon rank sum test with Holm method. ***p < 0.001. (D) R60F09-LexA and R73D06-GAL4 co-label AMBs. The yellow dot square indicates the area shown in the lower row. The yellow arrowheads indicate the somas of AMBs. Genotypes: LexAop-mCD8GFP, UAS-mCD8RFP/+; R60F09-LexA/+; R73D06-GAL4/+. Scale bars, 50 μm. (E) GAL80 labeled by R60F09-LexA diminishes CsChrimson expression in AMBs labeled by R73D06-GAL4 co-labeling. The yellow arrowheads indicate the somas of AMBs. Genotypes: w; LexAop-rCD2RFP/R60F09-LexA, tsh-GAL80; R73D06-GAL4/UAS-CsChrimson (the upper row), w; LexAop-rCD2RFP/R60F09-LexA, tsh-GAL80; R73D06-GAL4/UAS-CsChrimson, LexAop-GAL80 (the lower row). Scale bars, 50 μm. (F, G) The number of backward/forward waves in 10 seconds before (OFF) or during (ON) optogenetic activation with CsChrimson. n = 18, 20 for each genotype. In the boxplot, the width of the box represents the interquartile range. The whiskers extend to the data point which is less than 1.5 times the length of the box away from the box, and the dot represent outlier. We assessed statistical significance by the Wilcoxon rank sum test with Holm method. ***p < 0.001. (H) Probability of animals showing rolling behavior in 10 seconds before (OFF) or during (ON) optogenetic activation with CsChrimson. We assessed statistical significance by the Fisher’s exact test. (TIF) [file pgen.1009120.s002.tif]

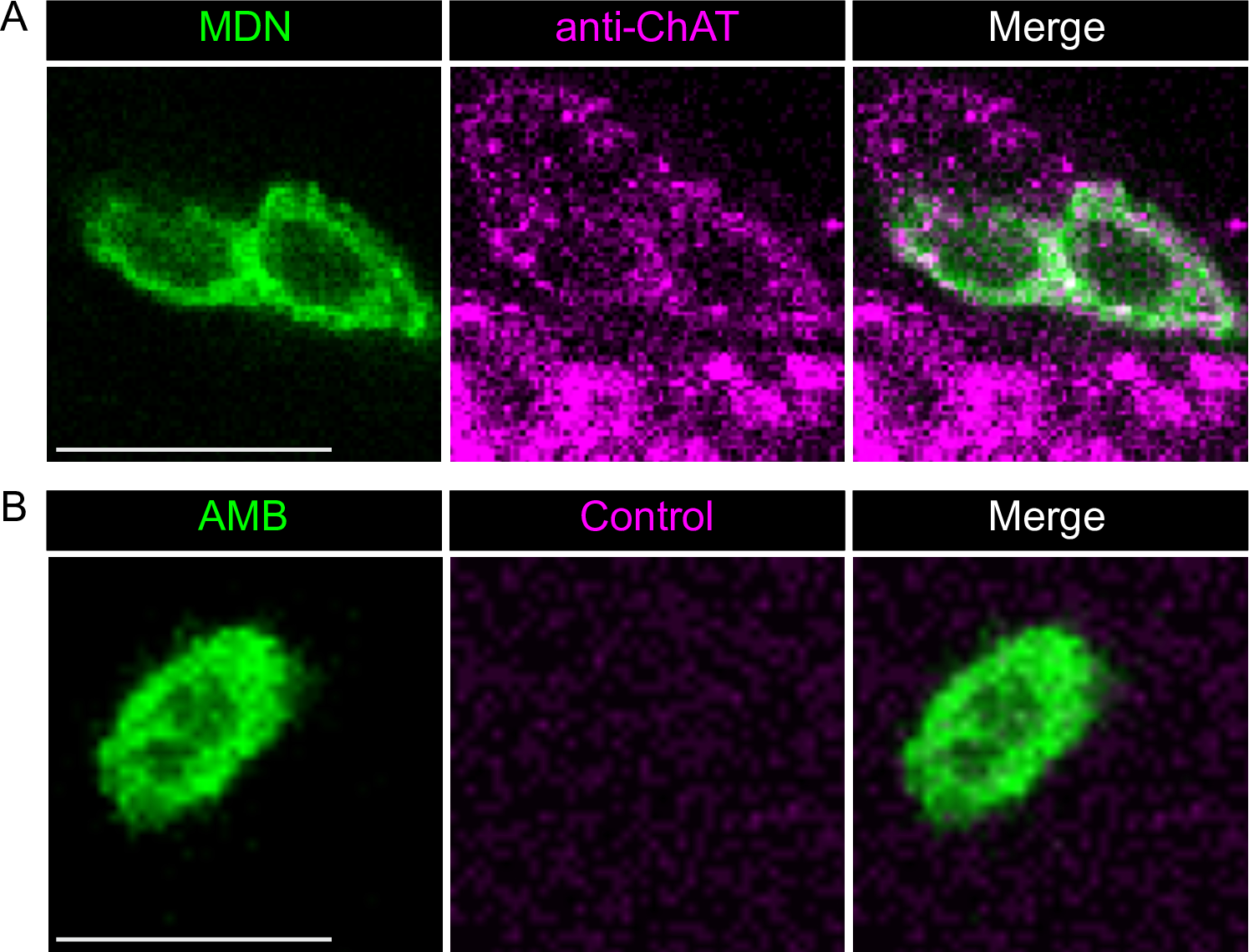

Supplement: S3 Fig — (A) Positive control for anti-ChAT. Anti-ChAT stained MDN somas that are reported to be cholinergic neurons in the previous study [7]. (B) Negative control of 2nd antibodies for immunohistochemistry. No detectable immunostaining was observed in the AMB somas without anti-ChAT antibody. Scale bars, 10 μm. (TIF) [file pgen.1009120.s003.tif]

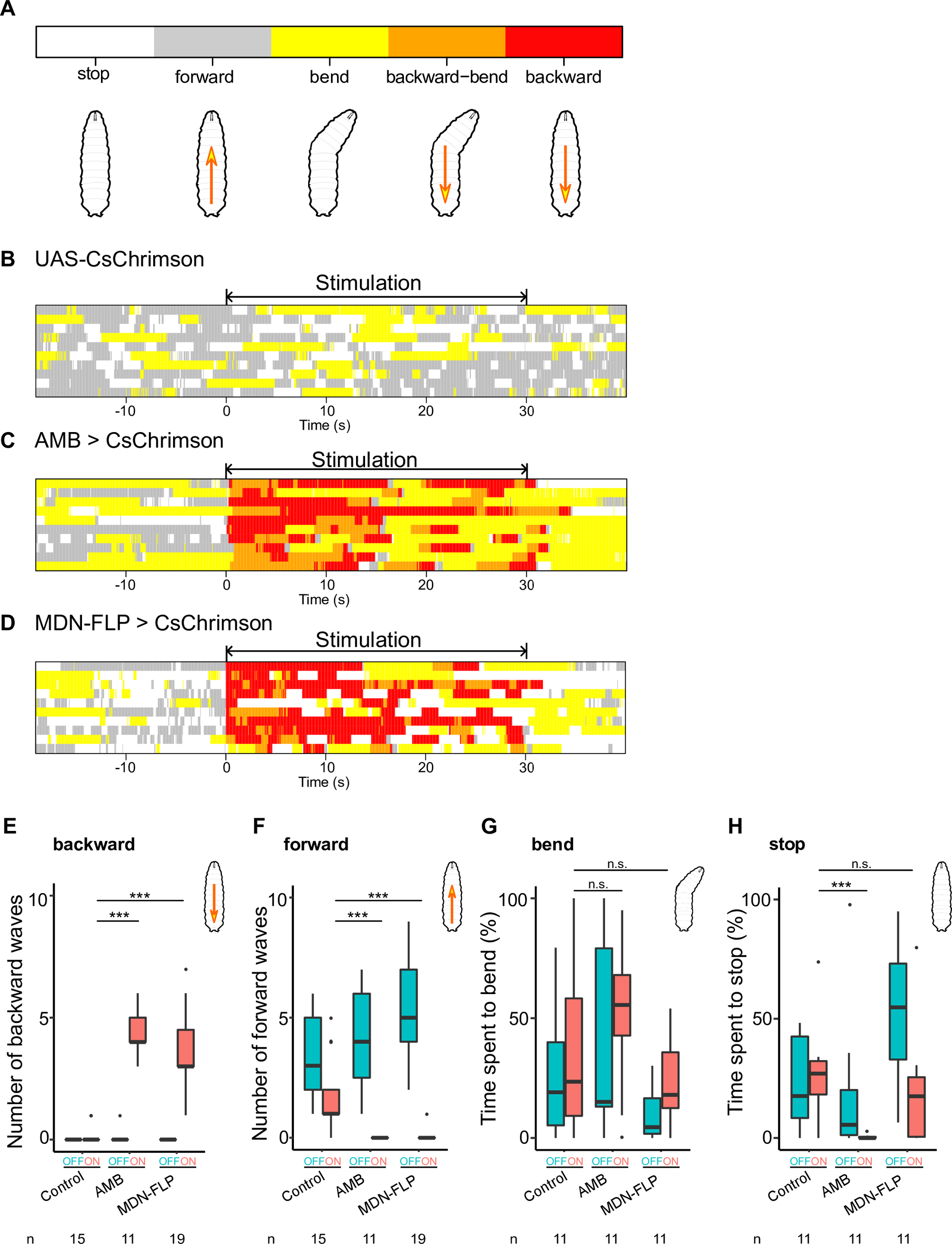

Supplement: S4 Fig — (A) Behavior events are color-coded: forward movement (grey), stop (white), bending (yellow), bending with backward locomotion (orange), and backward locomotion (red). (B-D) Behavior ethograms upon optogenetic stimulation of AMBs or MDNs. An animal expressing CsChrimson in either population was subjected to optogenetic activation for 30 seconds. Representative data from 10 different animals are shown for each genotype. (E-H) The number of backward/forward waves or percentage of time spent in a behavioral mode in 10 seconds before (OFF) and during (ON) optogenetic AMB activation with CsChrimson while silencing MDNs. In the boxplot, the width of the box represents the interquartile range. The whiskers extend to the data point which is less than 1.5 times the length of the box away from the box, and the dot represent outlier. We assessed the statistical significance by the Wilcoxon rank-sum test with the Holm method. ***p < 0.001. (TIF) [file pgen.1009120.s004.tif]

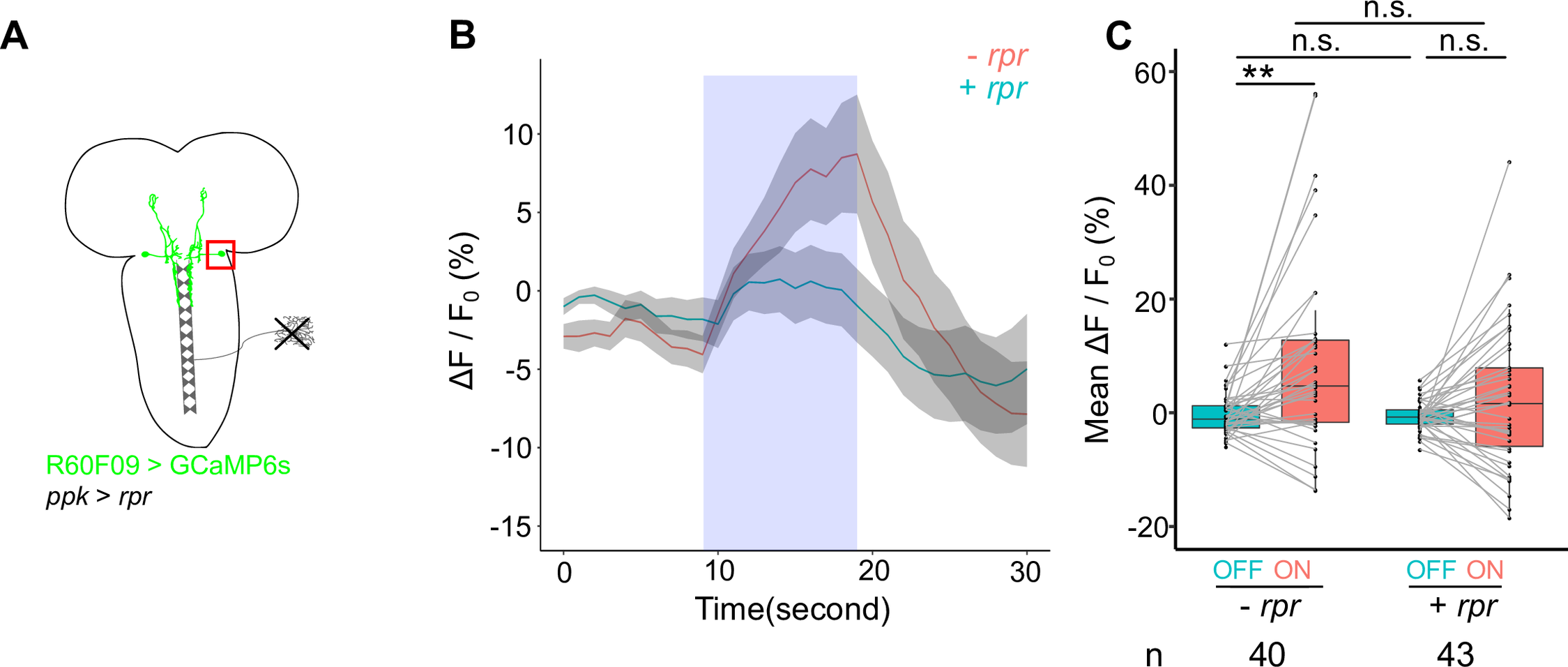

Supplement: S5 Fig — (A) A schematic view of Ca2+ imaging on AMB’s soma while ablating C4da neurons. Genotypes: UAS-rpr/+; R60F09-LexA, LexAop-GCaMP6s; ppk-GAL4/+ (+rpr); UAS-rpr/+; R60F09-LexA, LexAop-GCaMP6s; +/+ (-rpr). (B) Time series of Ca2+ responses in the soma of AMBs upon blue light irradiation. We applied stimulation in the period indicated by the blue band. Data are shown as the mean ± SEM. -rpr, n = 40; +rpr, n = 43. (C) Average of AMB ΔF/F0 values in 5 seconds before (OFF) or last 5 seconds during (ON) optogenetic activation. In the boxplot, the width of the box represents the interquartile range. The whiskers extend to the data point which is less than 1.5 times the length of the box away from the box, and the dot represent outlier. We assessed statistical significance by paired t test for paired samples and Welch’s two sample t-test for unpaired samples. **p < 0.01. (TIF) [file pgen.1009120.s005.tif]
